# Synthesis and characterization of a modified surface of SBA-15 mesoporous silica for a chloramphenicol drug delivery system

**DOI:** 10.1016/j.heliyon.2019.e02539

**Published:** 2019-10-01

**Authors:** Talib M. Albayati, Issam K. Salih, Haneen F. Alazzawi

**Affiliations:** a Department of Chemical Engineering, University of Technology, 52 Alsinaa St., PO Box 35010, Baghdad, Iraq; b Department of Chemical and Petroleum Industries Engineering, Al- Mustaqbal University College Hilla City PO Box 100, Babylon, Iraq

**Keywords:** Chemical engineering, Nanotechnology, Surface functionalization, Drug adsorption, Chloramphenicol, Drug delivery, Drug release, SBA-15, Nanomaterials, Materials synthesis, Surface chemistry

## Abstract

In this work, the potential of the modified SBA-15 surface was examined as a sorbent to load the drug from an aqueous solution; this was done using a post-synthesis function procedure. Several means were used to identify the material characterization before and after functionalization, such as X-ray diffraction (XRD), scanning electron microscopy (SEM), BET surface area, Fourier transform infrared (FTIR) spectroscopy and thermal gravimetric analysis (TGA). To obtain the effect of different variables on the efficacy of chloramphenicol drug load, batch adsorption experiments have been performed in a single adsorption system. These variables were the dosage of NH_2_-SBA-15 (10–120) mg, contact time (0–72 h) and initial concentration (10–120 mg/L). The results of these experiments showed the significant and active effect of the functional amino group in increasing the drug's load capacity. The results of these experiments showed that the functional amino group had a significant and active effect in increasing the drug's capacity. Also, the loading capacity is inversely proportional to the initial concentration, but directly proportional to the NH_2_-SBA-15 dose and contact time. The best results at 1 hour for the release were 41%. It was found that the load efficiency of chloramphenicol was 51%.

## 1 Introduction

Currently, injection and oral administration are the most-used methods of drug delivery for humans. But these ways have lower efficacy for some therapies [1]. New delivery systems are desired because some curative agents are poorly soluble or unstable. With some methods of administration, drugs must go through different physiological gateways before reaching the required site, consequently reducing the amount that reaches the targeted location. Failure to adequately deliver the proper dosage to the required site can result in lower drug efficiency [2]. Lowering the drug concentrations to be transported by employing nanostructure based on drug delivery vehicles with enhanced adsorption capability and controlled drug release properties can improve the drug's efficiency [3, 4, 5, 6, 7]. Consequently, mesoporous silica particles with large pore diameters are considered the perfect nominees for delivering high concentrations of different drugs by employing various templates to enhance encapsulation of large drug molecules [8, 9, 10, 11]. Furthermore, silanol groups may also be used with various organic groups to change surface properties that motivate suitable surface-drug reactions, which results in enhanced loading capability for drug molecules [12, 13, 14]. Mesoporous silica is believed to be a good nominee to deliver and release drugs because of favorable features that may help decrease adverse reactions and undesired side effects that many traditional drugs pose. Since their detection in the early 1990s [15], mesoporous silicates as MCM-41, MCM-48, and SBA-15 have attracted the attention of scientists as drug delivery vehicles [16] because of features such as high surface area (typically 1000 m^2^/g), high porosity (pore volumes 0.5–1.5 cm^3^/g), well ordered, tunable pores (typically 2–15 nm pore diameter) [17, 18, 19], and ‘‘non cytotoxic’’ properties [20, 21]. Furthermore, drug release rate is a problem that may be resolved by applying mesoporous silica in drug delivery [22]. Studies appear to show that the loading and releasing properties of a drug depend on the surface chemical nature, specific surface area, size, pore structure, and morphology of mesoporous silica particles [23, 24, 25, 26].

This work depicts chloramphenicol adsorption on SBA-15 functionalized surface. Chloramphenicol is an antibiotic used to treat bacterial infections. This includes as an eye ointment to treat conjunctivitis. It is applied orally or by injection to cure plague, meningitis, typhoid fever, and cholera [27]. The aim from this study is to investigate the influence surface functionalization on the adsorption and release of chloramphenicol.

In this work, a new method of producing effective adsorbents for chloramphenicol has been investigated using surface engineering modification to create highly exposed NH2 specific dispersible sites (3-aminopropyltriethoxysilane; APTES). No adsorption studies of Chloramphenicol antibiotic by Amino-functionalized SBA-15 in (H_2_N (CH_2_)_3_Si (OC_2_H_5_)_3_, were found in the literature. Hereon, it is described how the surface characteristics of NH_2_-SBA-15 are critical to determine the adsorption properties towards antibiotics.

## 2 Materials and methods

### 2.1 Chemicals

Pluronic “P123” tetraethylorthosilicate (TEOS, 98%), Hydrochloric acid (HCl), distilled water (H_2_O), toluene (C₆H₅-CH₃), 3-aminopropyltriethoxysilane (APTES), (H_2_N (CH_2_)_3_Si (OC_2_H_5_)_3_, 99%), potassium hydrogen phosphate (K_2_HPO_4_, 99%), potassium dihydrogen phosphate (KH_2_PO_4_, 99%)_,_ chloramphenicol (99%), that from Sigma Aldrich purchased. All chemicals were applied as obtained without additional treatment.

### 2.2 Synthesis of SBA-15

SBA-15 was prepared by using a standard technique discovered by Zhao et al. (1998) [28]: 6 g from the surfactant template P123 was dissolved in 45 g of distilled water. Then 180 g from 2M HCl was added to the previous solution at 35 °C until the surfactant was dissolved completely. Then 12.75 g of TEOS was gradually added to the surfactant solution and mixed for 20 h at 35 °C. The precipitated solution was then aged in sealed glass bottle for 24 h at 100 °C under static conditions. Then the white precipitate, which was allowed to cool to room temperature, was filtered, washed with distilled water, and then dried at standard conditions of 25 °C for 12 h. A white powder, SBA-15, was obtained when the surfactant was removed by calcinations for 6 h at 550 °C [29].

### 2.3 Surface functionalization

Amino-functionalized SBA-15 was synthesized by the method explained by Burke et al. (2009) [30]. At first, 1 g of calcined SBA-15 was dried for 3 hr at 100 °C. Then it was stirred under reflux with 40 ml of toluene and 10 ml of 3-aminopropyltriethoxysilane (APTES) for approximately 6 h. The suspension was then cooled, filtered, washed with toluene, and dried at 60 °C, resulting in the white powder amino-SBA-15.

### 2.4 Characterization

The X-ray diffractogram was used to find the crystalline structure, to identify crystalline phases and orientation, and to determine structural properties of the pure and functionalized SBA-15 with 2Ɵ in the range 0^o^ to 10^o^ with scan rate 2 (deg./min.). The Cu Kα λ = 1541Å was the source of X-ray radiation. The XRD test was done by X-Ray diffractometer (XRD-6000, Shimadzu, Japan). The SEM is a powerful method for analyzing the morphology and structure of the synthesized SBA-15 after and before functionalization. This was done by using a scanning electron microscope (SEM) (AIS2300C, South Korea). The total pore volume and specific surface area for SBA-15 before and after functionalization were obtained by using nitrogen adsorption isotherms (Brunauer, Emmett, and Teller method) on a surface area analyzer (Qsurf 9600, USA). The thermal gravimetric analysis (TGA) is an experimental method of thermal analysis which is used to find the incorporation of functional groups for functionalized SBA-15 and also to obtain information concerning thermal stability. The samples were heated under ambient pressure from room temperature to 650 °C with a rate of 10 °C/min. and analyzed by using the thermos gravimetric analyzer TG-DSC (STA PT1000, USA). The FT-IR instrument was used to analyze the functional groups grafted onto the SBA-15 and chemical bonds.

### 2.5 Chloramphenicol loading

Using 20 mg/l of chloramphenicol, a solution of chloramphenicol was manufactured. Then 50 mL of drug solution was mixed with 60 mg of NH2-SBA-15 and stirred for 72 h at room temperature. Using a UV analyzer, the loading of chloramphenicol was examined. The change in concentration was measured at different time intervals: (4, 8, 12, 24, and 48), and 72 h. And then solution was mixed with 60 mg of SBA-15 at room temperature, stirred for 24 h contact time. At different concentration ranges from 10-120 mg/l, the change in concentration was measured. Then SBA-15 was mixed with 20 mg/l of chloramphenicol concentration for 24 hr. To calculate the amount of chloramphenicol loaded, the absorbance values that obtained at 304 nm were used. According to Eq. (1) the loading percentage was calculated [31]:

(1) $$\%\;Loading=\frac{Weight\;of\;loaded\;drug}{Weight\;of\;NH2-SBA-15}\;\times 100\;$$

### 2.6 Chloramphenicol release

Using UV analysis the release of chloramphenicol was determined. This experiment can be explained as follows: using two different phosphate salts (K2HPO4 and KH2PO4) the phosphate buffer solution was prepared. To obtain the PH desired, the pH was set to 7.4 for the prepared solution in order. After that, 500 mL of PBS solution at body temperature was mixed with 200 mg of NH2-SBA-15 loaded with 124.08 mg/l of chloramphenicol. Within (6 h) By Eq. (2), the amount of the drug released from the NH2-SBA-15 molecules was determined according to change the concentration of the drug.

This experiment can be explained as follows: First, the phosphate buffer solution was prepared using two different phosphate salts (K_2_HPO_4_ and KH_2_PO_4_). The pH was set to 7.4 for the prepared solution in order to obtain the PH desired. After that, 200 mg of NH_2_-SBA-15 loaded with 124.08 mg/l of chloramphenicol was mixed with 500 mL of PBS solution at body temperature. The amount of the drug released from the NH_2_-SBA-15 molecules was determined by Eq. (2) within 6 h, according to change the concentration of the drug.

(2) $$\text{\% ​Release}=\frac{Weight\;of\;\;drug\;in\;solution}{Weight\;of\;drug\;in\;\;NH2-SBA-15}\times \;100\;$$

## 3 Results and discussion

### 3.1 Adsorbent characterization

#### 3.1.1 XRD analysis

**Fig. 1:**
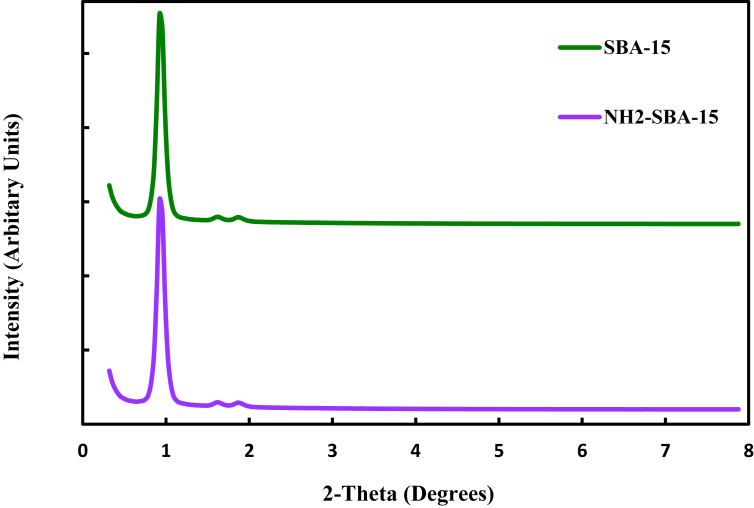
XRD patterns for SBA-15 and NH_2_–SBA-15.

The XRD patterns for SBA-15 and NH_2_–SBA-15 is shown in Fig. 1. Both mesoporous materials exhibit a single high-intensity peak (100) at 2Ɵ value of 0.96°, followed by two additional smaller peaks, at (110) and (200), at 2Ɵ lower than 2°, which confirms the formation of a hexagonal lattice of p6mm symmetry. Consequently, grafting the internal channels of mesoporous SBA-15 with the amino group did not affect the whole assembly of the ordered mesoporous silica. The XRD peak intensities of NH_2_–SBA-15 were basically minimal compared to SBA-15, which is possibly due to the fixing ligands on the outer surface of SBA-15 or the pore-filling effect of SBA-15 channels [32].

#### 3.1.2 FT-IR analysis

**Fig. 2:**
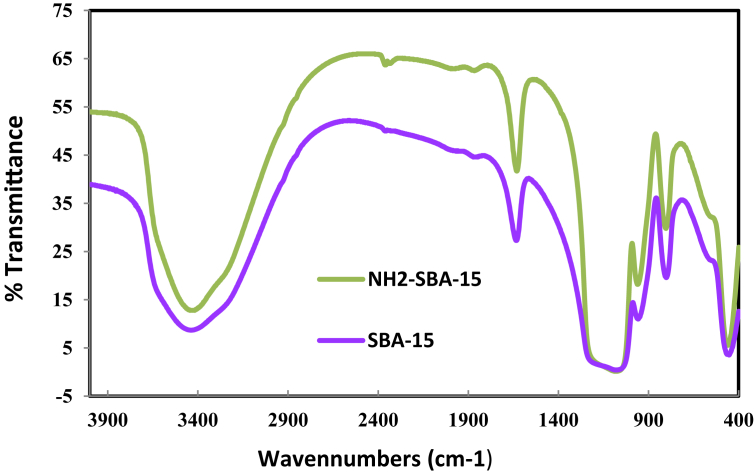
FTIR for SBA-15 & functionalized SBA-15-NH_2_.

The IR bands are related to silanol groups on the surface stretching vibrational mode, and they appear in the range 3740–3500 cm^−1^, as shown in Fig. 2. Furthermore, the NH bands are ordinarily existent at 3380–3310 cm^−1^, and because analysis isn't able to recognize those bands, the asymmetric NH_2_ bending becomes crucial to determining if an amine-group exists or not. Consequently, the band at around 1600 cm^−1^, which consists of two different peaks, may be attributed to asymmetric NH_2_ bending, which means that an amine-group is existent, as predicted. The band is attributed to Si–O–Si asymmetric stretching vibration at around 1100 cm-1, while the peak at around 800 cm^−1^ exists because of symmetric stretching vibration. In addition, the pure and functionalized samples own smaller peaks at around 400 cm^−1^, which may be attributed to the distortion modes of Si–O–Si. Also, the band at around 950 cm^−1^ may be caused by the Si-OH bending [33].

#### 3.1.3 SEM analysis

**Fig. 3:**
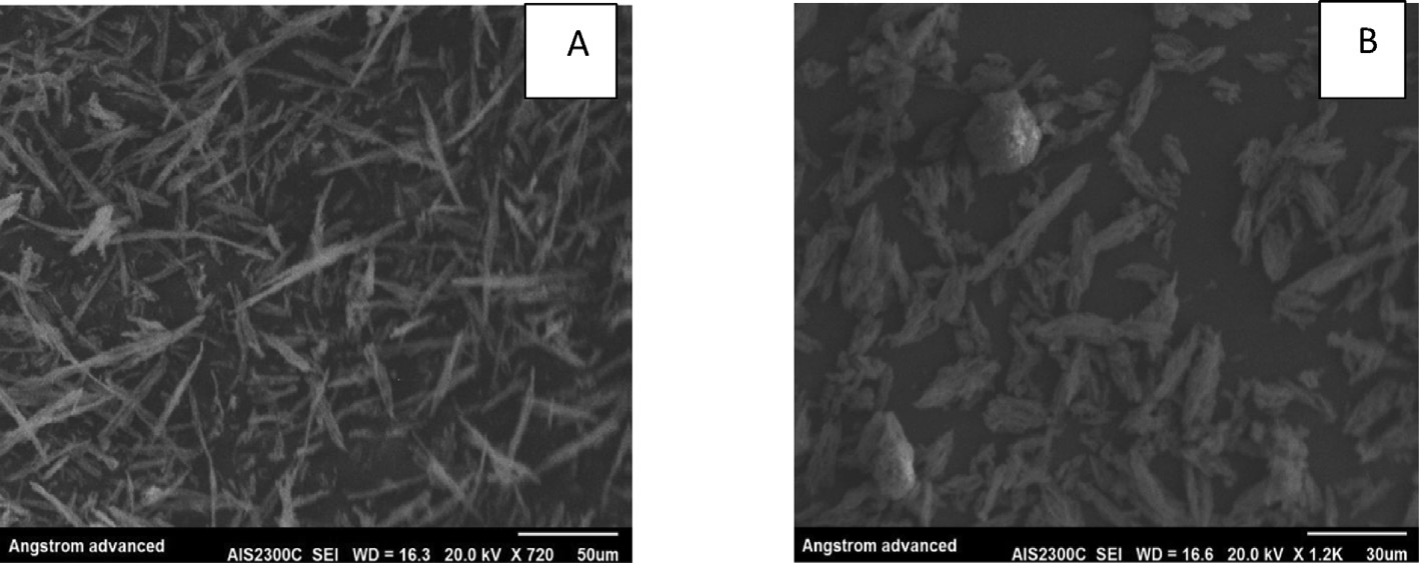
SEM images for (A) pure SBA-15 and (B) functionalized NH2 – SBA-15.

The morphologies of SBA-15 and NH_2_-SBA-15 are shown in Fig. 3. According to SEM images, the particles were observed to be small rods for both samples, which mean the samples maintained their structural properties [34].

#### 3.1.4 TGA analysis

**Fig. 4:**
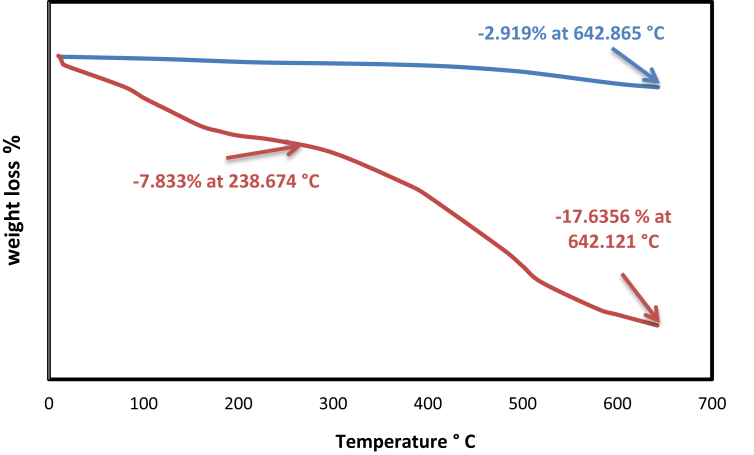
Thermal gravimetric analysis for SBA-15 and NH_2_-SBA-15.

The weight loss of thermal gravimetric analysis is plotted in Fig. 4 for the samples of SBA-15 and NH_2_-SBA-15, which were heated from room temperature to 650 °C, at a rate of 10 °C/min. The thermal gravimetric curve showed at 643 °C that for pure SBA-15, the mass losses were 3%, which can be considered a negligible value due to the very small rate of mass loss at this temperature. The calcined SBA-15 indicates complete removal of the surfactant during the calcinations process, and it proves the thermal stability of SBA-15. This process was done at a temperature lower than the one required reaching the melting point of silica, and for NH_2_-SBA-15, there is a weight loss in the temperature range of 200–300 °C was due to the decomposition of organic moiety as a result of amino functional group loading on the surface of SBA-15 and the total weight losses was 18% at 642 °C [33].

#### 3.1.5 BET surface area analysis

The prepared SBA-15 had a BET surface area of 675 m^2^/g and pore volume of 0.7908 cm³/g, and for functionalized SBA-15, the BET surface area is 169 m^2^/g and pore volume of 0.028 cm³/g. These variables agree with those in the literature [35]. Large BET surface area for the hexagonal channels was seen in the SBA-15 samples. The existence of functional groups at the mesoporous matrix surface promotes drug adsorption. The results show that there is a decrease in pore volume and surface area after functionalization. This confirms that the functional group is located not only on the outer surface but also inside the mesoporous channels [36].

### 3.2 Chloramphenicol loading

#### 3.2.1 Effect of contact time

**Fig. 5:**
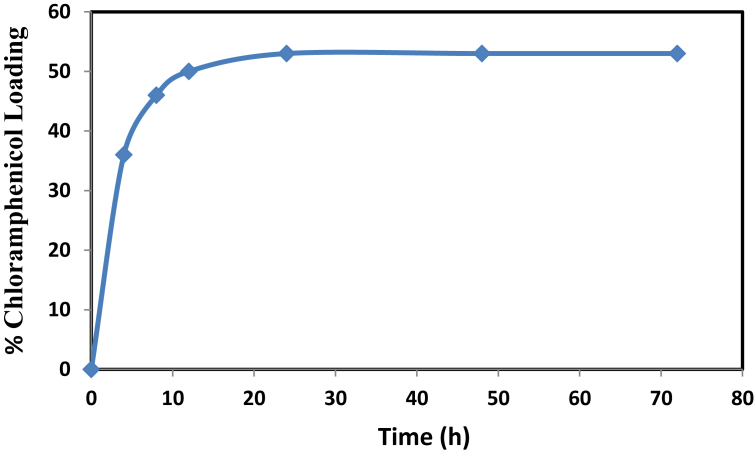
Effect of contact time on chloramphenicol loading at initial concentration of chloramphenicol 20 mg/L and dosage of NH_2_-SBA-15 = 60 mg.

The relationship between the load capacity of chloramphenicol and contact time is shown in Fig. 5. This figure indicates that the time required to reach equilibrium absorption is directly proportional to the loading capacity. The experiments showed that in the first few hours, from 0 to 4 h there was a quick increase of chloramphenicol capacity of load due to the larg amount of mesoporous particle surface area that was ready for loading. Then, from 4 to 24 h chloramphenicol drug loading lessened with time, and this was due to the decreasing availability of mesoporous particle surface area for loading. However, the curve of drug loading capacity became almost horizontal after 24 h, which indicates that the surfaces of mesoporous particles were filled, (saturated) or, in other words, had reached the equilibrium adsorption. The best loading time was the first 24 h. Loading was done up from 24 to 72 h. It is clear that 2% more than that done in the first 24 h, as shown in Fig. 5 [37].

#### 3.2.2 Effect of initial concentration

**Fig. 6:**
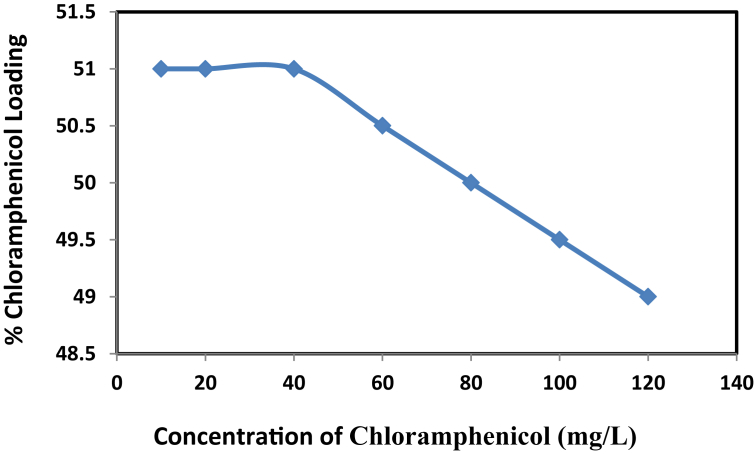
Effect of concentration on chloramphenicol loading at contact time 24 h and dosage of NH_2_-SBA-15 = 60 mg.

The effect of initial concentration was investigated on adsorption conduct for chloramphenicol. The relationship between these two variables is shown in Fig. 6.

When the active site of the adsorbent is able to adsorb 51% of the analytic when its concentration is up to 20 mg/l was reached a state of equilibrium, the curve became almost horizontal because no more sites of adsorption were available to be filled by the drug. Then, the relationship between the concentration and load capacity for chloramphenicol was inversely proportional: as long as the adsorption reached its limit, the load capacity decreased proportionally with increasing the concentration drug, or “fill out the number of active sites.” Then, The best concentration and maximum load capacity of chloramphenicol was 20 mg/l and 51% respectively [38].

#### 3.2.3 Effect of dose

**Fig. 7:**
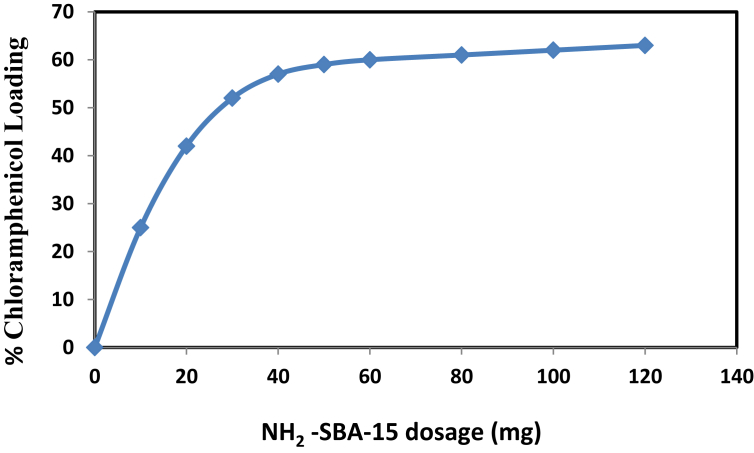
Effect of NH_2_-SBA-15 dosage on chloramphenicol loading at initial concentration of chloramphenicol 20 mg/L and at contact time 24 h.

The effect of NH_2_-SBA-15 dosage was investigated for chloramphenicol, and the results are shown in Fig. 7. After studying chloramphenicol drug concentration effect and the contact time, the study of NH2-SBA-15 dosage effect was done, according to the experimental work and after obtaining the best values. The best concentration was assumed: 20 mg/l. The best value was also used as constant for contact times: 24 h. The results show that the drug load capacity increased proportionally with the dosage of NH2-SBA-15 until it reached equilibrium adsorption, and then the curve became horizontal. The best dosage value can be taken at the initiation of this change: 40 mg. Increasing the load capacity of the drug is proportionally associated with an increase in the adsorbent dose because the increase in the adsorbent dose means that more mesoporous molecules are available in the solution and more surface areas are available for loading, resulting in more drug being loaded [39].

### 3.3 Chloramphenicol release

**Fig. 8:**
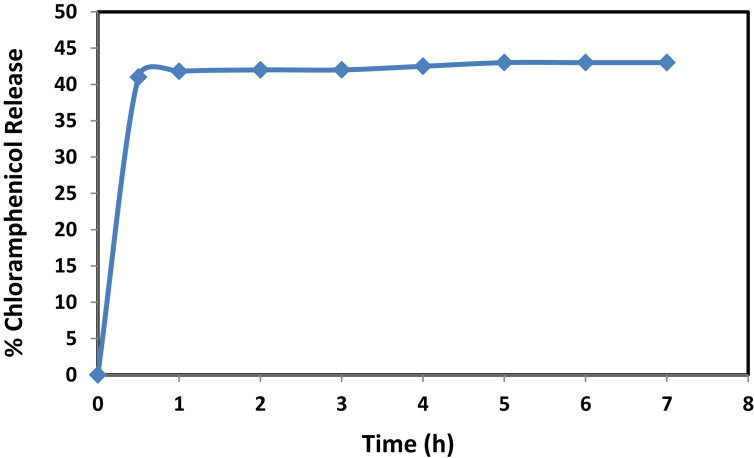
The chloramphenicol release profile of NH_2_-SBA-15 sample.

With the 3 amino propyl groups, it was established that the surface of SBA-15 becomes hydrophilic. Consequently, the reaction between mesoporous silica and the drug causes variation in terms of the release rate and loading capacity, as shown in Fig. 8. The release of drugs may be controlled by delivery with NH_2_-SBA-15. The maximum release is within 30 minutes and at 41%, which can continue for the next 6 h. This could have been because of the driving force effect (concentration variance), where the drug was transferred from a higher concentration "from adsorbent" to a lower concentration of PBS ′phosphate buffer solution", and the release (desorption) increased over time until reaching equilibrium at 1 h. Therefore, NH_2_-SBA-15 is a promising nominee for drug loading and control of release [40].

### 3.4 Kinetic model of drug release

The Weibull models, Korsmeyer-Peppas and non-Fickian, were carefully applied to examine the kinetic release process [41]. The non-Fickian model followed the diffusional release process in Eq. (3) under the condition (n > 1). When this equation was applied, the value of (n) was less than one, which doesn't comply with the boundary conditions.

(3) $$\text{Y ​}=\text{ ​}{\text{at}}^{\text{n}}$$

where: Y is the absolute cumulative percentage amount of drug released and a is a constant reflecting the design variables of the system.

The Korsmeyer-Peppas model format was used to check the drug's release in the following equation under the condition that Mt/M∞ must be less than 0.6. When applied this equation, the value of (Mt/M∞) was more than (0.6), which doesn't comply with the boundary conditions.

(4) $$\text{Mt/M}\infty \text{ ​}=\text{ ​}{\text{kt}}^{\text{n}}$$

Where: Mt and M∞ describe the amount of the drug released after time t and the initial amount of the drug in the carrier, respectively, Mt/M∞ is a fractional release of drug, k is a constant incorporating structural and geometric characteristic of drug formulation, n is the release exponent, and t represents duration time of release.

By the Weibull model in the following equation, the cumulative release percentage from the drug may be checked. Where R^2^, the (coefficient of determination), must be equal to or higher than (0.96).

(5) Ln[ln1/(1−f)]=mlnt−lnto

**Fig. 9:**
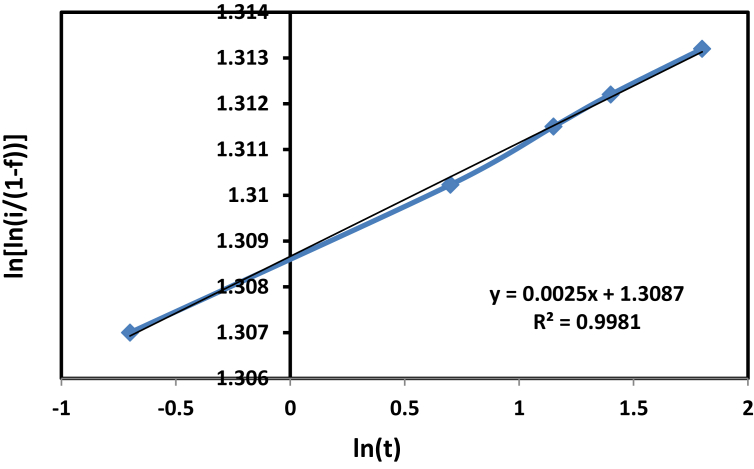
The Release kinetic model of chloramphenicol drug delivery system.

The value of R^2^ was more than (0.96) so this equation was applied, which corresponds to the boundary conditions, so it was effectively used to emulate the release of the drug as detailed below. The cumulative quantity of the drug released is f. The Weibull shape parameter is m, which shows the effect of non-released drug mass on the release rate, which is (0.0025), and (ln t_o)_ be the intercept, which is 1.3875. Also, t is the release, or dissolution, time. Ln [ln 1/(1 − f)] is proportional to ln t, and plotting (ln t) versus ln [ln 1/(1 − f)] leads to a line of slope (m). In our particular case are shown in Fig. 9, the curves fitted to the experimental points by Eq. (5). A good fit of data to the modulus was achieved with a determination coefficient R^2^ of 0.9981.

## 4 Conclusion

The synthesis of mesoporous silica NH_2_-SBA-15 was obtained by a controlled post-grafting method. Characterization techniques such as XRD, SEM, BET surface, FTIR, and TGA proved the successful preparation of pure mesoporous material SBA-15 and modified surface of NH_2_-SBA-15. The post-grafting process allowed the manufacture of functional mesoporous NH_2_-SBA-15 for drug delivery, which displayed appropriate loading capacity to enhance the drug's therapeutic efficacy. The results of the best load capacity were 51%, and the results for release experiments were an optimum time of half an hour and with the value of 41.35%.

The diffusion equation was compared with the results of drug release with Weibull models, Korsmeyer-Peppas and non-Fickian, but the results didn't comply with the boundary conditions—except for that of the Weibull model. Our technology should result slow release rate for the chloramphenicol drug molecules and in high loading capacity, As well making mesoporous SBA-15 a promising carrier for a drug delivery system. We believe that the results of our study may also lead to a wide range of future drug delivery implementations.

## Declarations

### Author contribution statement

Haneen F. Alazzawi: Conceived and designed the experiments; Performed the experiments.

Issam K. Salih: Analyzed and interpreted the data.

Talib M. Albayati: Contributed reagents, materials, analysis tools or data; Wrote the paper.

### Funding statement

This research did not receive any specific grant from funding agencies in the public, commercial, or not-for-profit sectors.

### Competing interest statement

The authors declare no conflict of interest.

### Additional information

No additional information is available for this paper.
